# Very-early-onset Inflammatory Bowel Disease in an Infant with a Partial *RIPK1* Deletion

**DOI:** 10.1007/s10875-024-01707-8

**Published:** 2024-04-27

**Authors:** Ceyda Tuna Kırsaçlıoğlu, Alexandra Frohne, Zarife Kuloğlu, Isidora Kristofersdottir, Engin Demir, Cansu Altuntaş, Zehra Şule Haskoloğlu, Fatma Nazan Çobanoğlu, Tanıl Kendirli, Halil Özdemir, Zeynep Birsin Özçakar, Berna Savaş, Figen Doğu, Aydan İkincioğulları, Kaan Boztug, Aydan Kansu

**Affiliations:** 1 Department of Pediatrics, Division of Pediatric Gastroenterology, Hepatology and Nutrition, Ankara University School of Medicine, Ankara, Türkiye Turkey; 2 St. Anna Children’s Cancer Research Institute (CCRI), Vienna, Austria; 3 Department of Pediatrics, Division of Pediatric Immunology and Allergy, Ankara University School of Medicine, Ankara, Türkiye Turkey; 4 Department of Pediatrics, Division of Pediatric Pulmonology, Ankara University School of Medicine, Ankara, Türkiye Turkey; 5 Department of Pediatrics, Division of Pediatric Intensive care, Ankara University School of Medicine, Ankara, Türkiye Turkey; 6 Department of Pediatrics, Division of Pediatric Infectious Disease, Ankara University School of Medicine, Ankara, Türkiye Turkey; 7 Department of Pediatrics, Division of Pediatric Nephrology and Rheumotology, Ankara University School of Medicine, Ankara, Türkiye Turkey; 8 Department of Pathology, Ankara University School of Medicine, Ankara, Türkiye Turkey; 9 Ludwig Boltzmann Institute for Rare and Undiagnosed Diseases, Vienna, Austria; 10 CeMM Research Center for Molecular Medicine of the Austrian Academy of Sciences, Vienna, Austria; 11 Department of Pediatrics and Adolescent Medicine, St. Anna Children’s Hospital, Medical University of Vienna, Vienna, Austria; 12 Department of Pediatrics and Adolescent Medicine, Medical University of Vienna, Vienna, Austria

**Keywords:** Very-early Onset Inflammatory Bowel Disease, Immunodefciency, Inflammation

## Abstract

The monogenic causes of very-early-onset inflammatory bowel disease (VEO-IBD) have been defined by genetic studies, which were usually related to primary immunodeficiencies. Receptor-interacting serine/threonine-protein kinase-1 (RIPK1) protein is an important signalling molecule in inflammation and cell death pathways. Its deficiency may lead to various clinical features linked to immunodeficiency and/or inflammation, including IBD. Here, we discuss an infant with malnutrition, VEO-IBD, recurrent infections and polyathritis who has a homozygous partial deletion in *RIPK1* gene.

## Introduction

Inflammatory bowel disease (IBD) is a collective term for a group of chronic inflammatory disorders of the digestive tract. The etiology involves both genetic and environmental factors, though in rare cases, IBD can be caused by an underlying monogenic immunodeficiency. Very-early-onset (VEO) IBD is defined by a disease onset before six years of age [1] and characterized by a higher proportion of monogenic cases as compared to later-onset forms (an estimated 15–20% of VEO-IBD) [2]. So far, more than 70 genes have been linked to monogenic VEO-IBD [3, 4]. Patients with VEO-IBD generally have a poor prognosis and are unresponsive to treatment, except for certain immunodeficiencies [3–6]. It is critical to identify underlying genetic abnormalities in order to evaluate treatment options. If the underlying problem only affects cells originating from the hematopoietic system, it can be treated with allogeneic hematopoietic stem cell transplantation (HSCT), but intrinsic defects in epithelial or stromal cells do not recover with HSCT and require additional treatments [7–9].

Receptor-interacting serine/threonine-protein kinase-1 (RIPK1) is a critical molecule in cell death and inflammatory pathways. RIPK1 deficiency has previously been reported in few patients presented with VEO-IBD, recurrent infections, combined immunodeficiency or autoinflammation syndrome [7–9]. Here, we report an infant who presented with malnutrition, recurrent infections (pneumonia, otitis, sepsis, oral moniliasis), polyarthritis and infantile IBD with perianal involvement. He was found to have a homozygous p.Glu148Gln variant in Mediterranean fever gene *(*MEFV innate immunity regulator, pyrin*)*, a 22.7 kb-deletion comprising the last four exons of *RIPK1* as well as the first exon of the adjacent *BPHL* (biphenyl hydrolase-like) gene. To the best of our knowledge, this is the first report of a patient with a deletion involving *RIPK1* and *BPHL*.

## Case Report

**Fig. 1:**
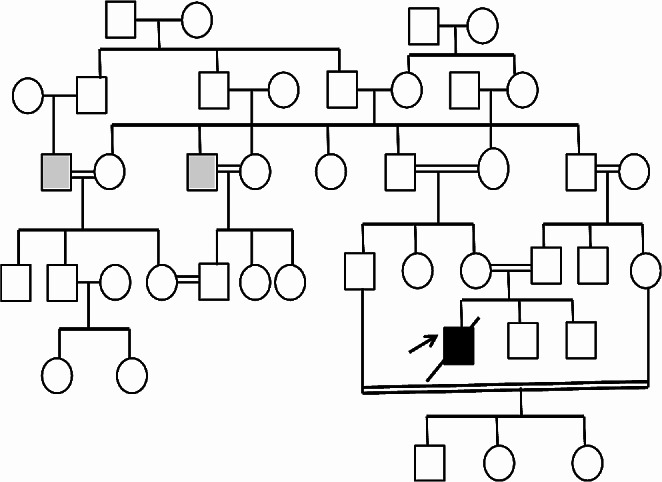
Pedigree of the patient. Grey box: Behçet Disease. Black arrow: The patient with RIPK1 deficiency

**Fig. 2:**
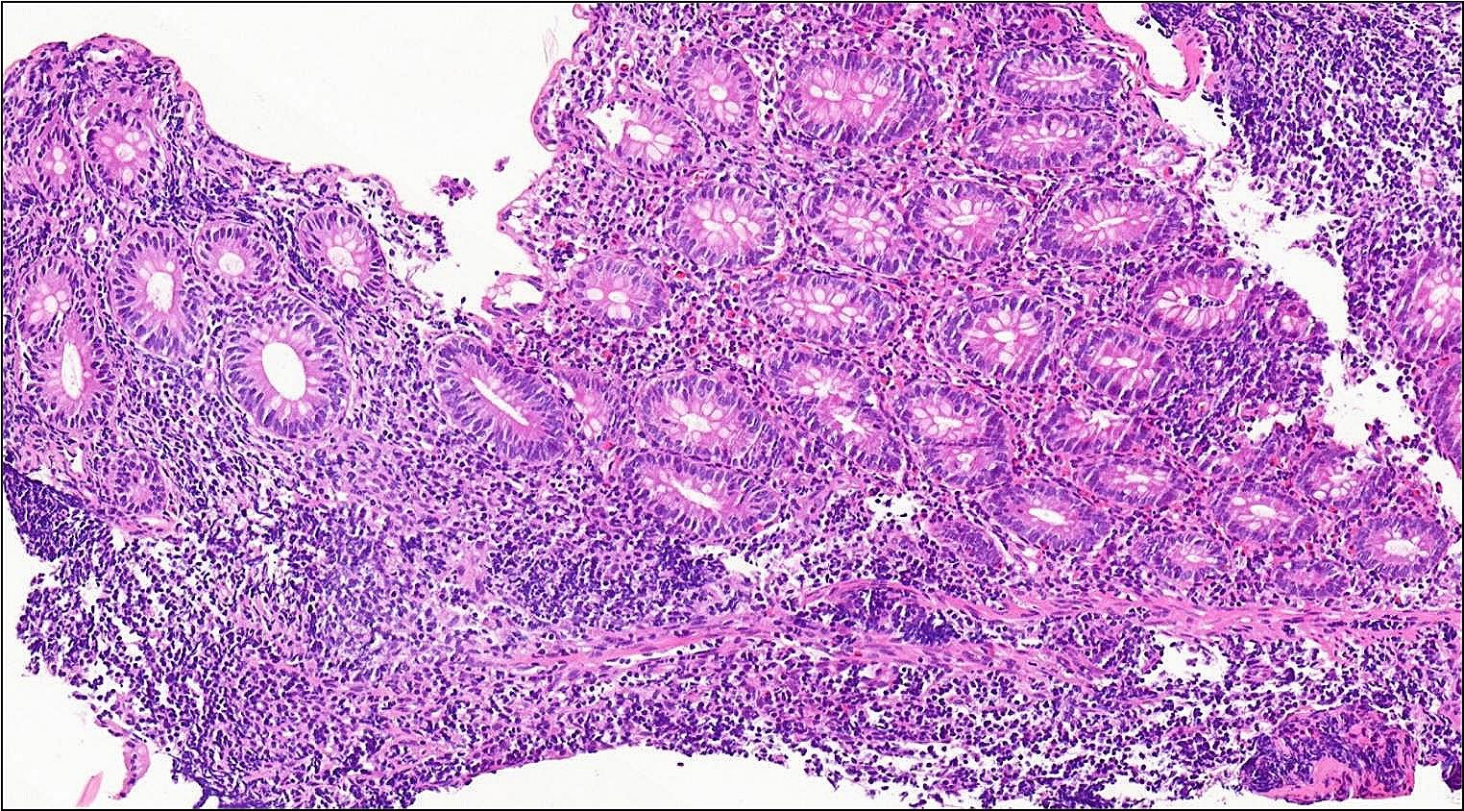
Colon mucosa is showing active chronic inflammation with cyrptitis and faint crypt distortion with focal goblet cell depletion (H&E, x15,8)

A nine-month-old boy was admitted to our hospital with fever, swelling of the hands, and watery, mucoid, and intermittently bloody diarrhoea (9–10 times/day). Diarrhoea had been present since birth. He had been hospitalized five times due to recurrent lower respiratory tract infections, recurrent otitis accompanied by intermittent arthritis in the hands and/or feet and acute gastroenteritis characterized by watery, mucoid, and intermittently bloody stools. Although he had normal weight gain velocity in the first six months of his life, it was insufficient (100 g/month) in the last three months. His parents were first-degree relatives, and the patient had third-degree relatives with Behçet’s Disease (Fig. 1). The physical examination revealed acute moderate malnutrition (weight and length for age z-score − 2.9 and − 1.5, respectively, and weight for length z-score − 2.8), long and curly eyelashes, oral moniliasis, high palate, an aphthous ulceration on the soft palate, bilateral coarse breath sounds and rhonchi, swelling and tenderness on the 3rd and 4th metacarpophalangeal and proximal interphalangeal joints of both hands. His laboratory tests revealed hypochromic microcytic anemia (Hemoglobin: 8.1 g/dL, mean corpuscular volume: 67 fL), thrombocytosis (669 000/mm^3^), hypoalbuminemia (3 g/dL), and elevated C-reactive protein [73.9 mg/L (N: 0–5 mg/L)]. Erythrocytes and leucocytes were positive in stool examination. Microbiological tests for cryptosporidium PCR and other diarrheal agents were negative. Sweat chloride test and thyroid function tests were normal. Advanced immunological work-up revealed normal peripheric blood leucocytes, lymphocyte activation with phytohemagglutinin (PHA) and oxidative burst test, except for decreased numbers of CD4^+^ lymphocytes and natural killer cells (Table 1). Immunoglobulin levels were in normal ranges (Table 1) and specific IgE for cow’s milk and egg yolk were negative. Abdominal ultrasonography and upper gastrointestinal endoscopy were normal, but colonoscopy revealed small aphthous ulcers, hyperemia, oedema, fragility on ileocecal valve and dominantly on distal colonic mucosa. Histopathology revealed mild esophagitis, *Helicobacter pylori* negative chronic non-atrophic gastritis, mild active colitis with cyrptitis, ulceration and faint cyrpt distortion in distal colonic mucosa (Fig. 2). Cytomegalovirus PCR was negative in blood and tissue examination. Small bowel follow-through radiologic examination was normal. Computed tomography of the chest revealed slight central ground-glass opacity and centriaciner micronodules on the upper lobes of bilateral lungs. Bronchoscopy was normal except for white-coloured mucoid secretion coming from both main bronchi and very mild stenosis of right bronchus intermedius. Bronchoalveolar lavage culture was positive for *Klebsiella pneumonia*.

**Table 1:** The immunological work-up of the patient

	Absolute value (%)	Reference range due to age (%)
White blood cell count (cells/mm^3^)	11,000	6000–17,500
Total neutrophil count (cells/mm^3^)	5870 (53.3)	1500 to 8500 (15–45)
Total eosinophil count (cells/mm^3^)	190 (1.7)	180–510 (1–4)
Total lymphocyte count (cells/mm^3^)	3700 (33)	3000–10,000 (45–75)
Lymphocyte subsets (cells/mm^3^)		
CD3 + CD16-56-	2146 (58)	2400–8100 (51–79)
CD3 + CD4+	296 (8)	1400–5200 (31–54)
CD3 + CD8+	1517 (41)	600–3000 (10–31)
CD3-CD16 + 56+	259 (7)	200–1800 (5–23)
CD4 + 45RA+	15 (5)	1200–5600 (25–45)
CD4 + 45RO+	12 (4)	300–1400 (6–21)
CD19+	1258 (34)	500–3600 (14–44)
HLA DR	779 (62)	200–3100 (15–48)
TCR γ/δ	370 (10)	(< 5)
CD4 + CD45RA + CD31+ (RTE)	(44)	(> 50)
**Lymphocyte proliferation tests**		
Response to PHA CD3 + 25+	54%	37–57%
CD3 + 69+	55%	57–69%
**Immunoglobulin levels**		
IgG (mg/dl)	894	463–1006
IgA (mg/dl)	51	17–69
IgM (mg/dl)	61	46–159
Total IgE (IU/ml)	9	< 15
**Serum antibody response**	Anti-HBs positive	
**Oxidative Burst Test**	Normal (MFI:138)	

Recurrent severe infections, growth retardation, VEO-IBD, reduced CD4 + T cells and consanguineous marriage suggested that a primary immunodeficiency may be the underlying cause, and genetic analysis was planned in order to identify the underlying defect. Rheumatological examination revealed normal anti-nuclear antibody, IgD, complement levels, a negative pathergy test but a homozygous p.Glu148Gln mutation (E148Q) in exon 2 of the *MEFV* gene by direct sequencing analysis of the PCR-restricted fragment length polymorphism (RFLP) protocol. Ophthalmological examination was normal. There were no specific findings indicative of a particular disease in metabolic tests. Extensively hydrolysed formula, methylprednisolone (2 mg/kg/day), mesalamine, colchicine, ibuprofen, azathioprine treatment and trimethoprim-sulphamethoxazole and fluconazole prophylaxis were given. Ibuprofen was ceased when arthritis resolved.

**Fig. 3:**
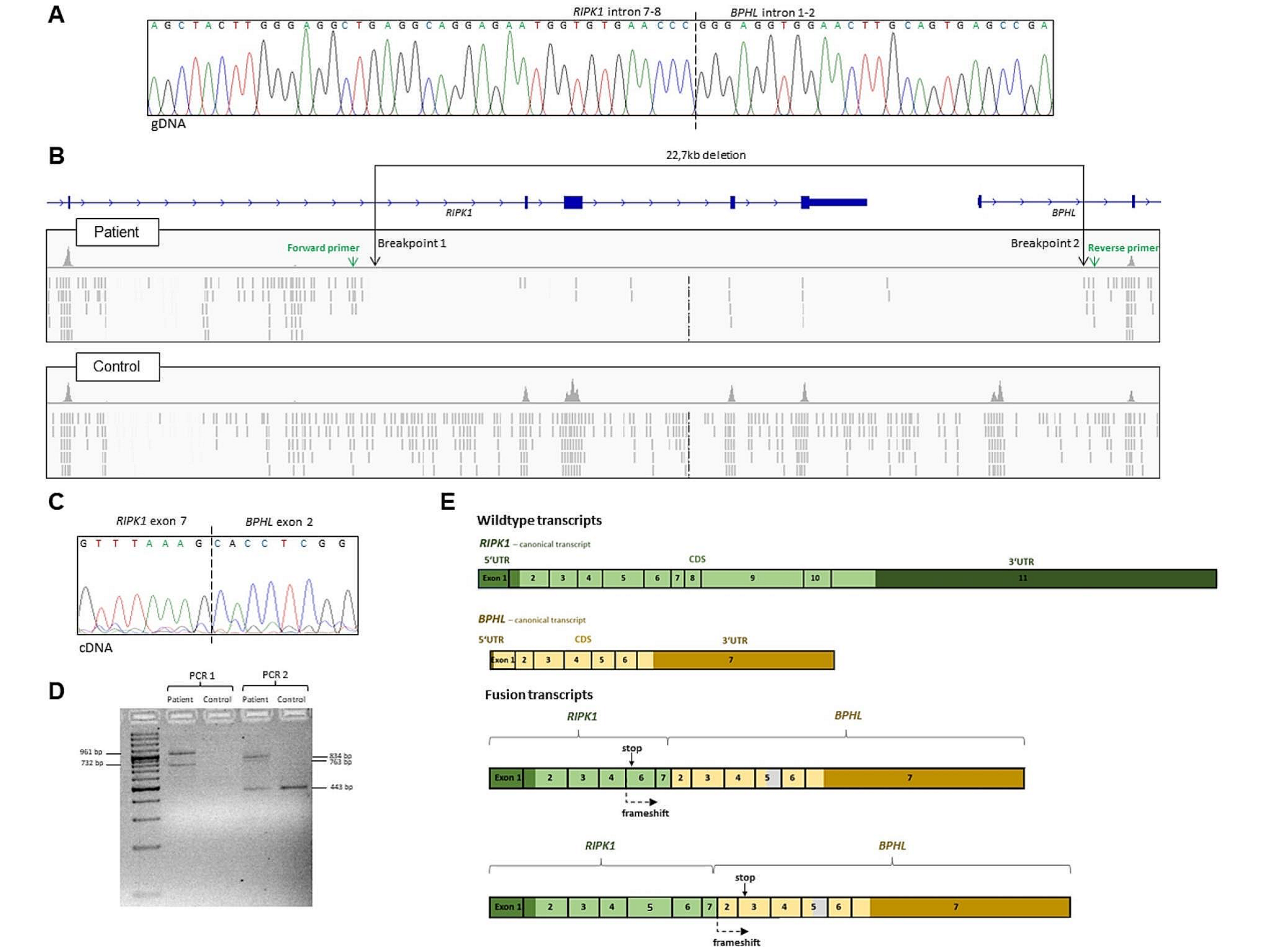
Chromatogram and Integrative Genome Viewer (IGV) visualization of the alignment. **(A)** Amplification of the genomic area carrying the deletion revealed the precise breakpoints of the deletion. Primers: 5’TGAGTTGGAGATTGGGGTGC3’ (forward) and 5’TTATGGGTGCCGTACAGGTG3’ (reverse). **(B)** The IGV coverage tracks of the patient and control exomes illustrate the position of the homozygous 22,726 bp deletion encompassing exons 8–11 and exon 1 in *RIPK1* (NM_001354930.2) and *BPHL* (NM_004332.4), respectively. The deletion was called from whole-exome data based on coverage values using the ExomeDepth software [10]. Since the breakpoints were located outside the targeted regions, their exact position could not be inferred from the whole-exome data alone. As illustrated by the IGV screenshot, the density of off-target reads mapped to the introns 7–8 (*RIPK1*) and 1–2 (*BPHL*) were visibly sparser in the patient compared to controls. Primers were designed based on the assumption that the breakpoint was located near the points where the read density starts to decrease (primer binding sites are indicated with green arrows). **(C)** Chromatogram showing the breakpoint of the cDNA fusion transcript between *RIPK1* and the following gene *BPHL*. cDNA was synthesized from mRNA from B-cell lines derived from the patient and a healthy control. PCR and Sanger sequencing showed that the deletion leads to a fusion transcript between *RIPK1* and *BPHL* in the patient. **(D)** Gel electrophoresis of the products from PCR 1 (primers binding in *RIPK1* exon 1 and *BPHL* exon 3; forward:5‘GGAAGGTGTCTCTGTGTTTCCA3’, reverse: 5‘GAGGTCCAAAATCAGTCTCTCCA3‘) and PCR 2 (primers binding in *RIPK1* exon 6 and *BPHL* exon 7; forward: 5‘GCTCTGCTGGGAAGCGAAT3‘, reverse: 5‘GGTTGTGTTTGCCTTCTGGC3‘). Sanger sequencing showed that the multiple bands correspond to different splice variants, caused by skipping of *RIPK1* exon 5 (PCR 1, 732 bp product) and the partial skipping of *BPHL* exon 5 (PCR 2, 763 bp product). The 443 bp band in PCR 2 is due to unspecific amplification of *ABCB8* cDNA in both the patient and the control subject. In the healthy donor, no other products were amplified due to the absence of a fusion transcript. **(E)** Illustration of the wildtype transcripts and the mutant fusion transcripts in the patient. All transcripts identified in the patient are predicted to lead to a frameshift and premature stop in either *RIPK1* exon 6 or *BPHL* exon 3, depending on whether or not *RIPK1* exon 5 is skipped. In a proportion of the transcripts, part of *BPHL* exon 5 is skipped (indicated in grey). Since *RIPK1* exon 5 and *BPHL* exon 5 were not included in the same amplicons, we do not know if the partial *BPHL* exon 5 skipping occurred in the transcripts including *RIPK1* exon 5, excluding *RIPK1* exon 5, or both

After one month of high-dose steroid treatment, there was no improvement in his clinical and laboratory findings and no weight gain. He was hospitalised for three times in the following six months due to lower respiratory tract infection, fever and diarrhoea. Therefore, immunosuppressive treatment was ceased as there was no beneficial effect. After one month, he was hospitalized for vomiting, diarrhoea, perianal abscess and perianal fistula, maculopapular rash, tenderness and limitation in the movement of the left arm. Total parenteral nutrition and broad-spectrum antibiotics were given, and drainage of the perianal abscess and fistulectomy were performed. He developed catheter-related *Klebsiella pneumonia* and *Enterococcus feacalis* infection, complicated with disseminated intravascular coagulation and intracranial haemorrhage. He was followed in the intensive care unit for five months under supportive treatment including mechanical ventilation, but unfortunately, he died at the age of two years due to *Acinetobacter boumanni* sepsis and multiorgan failure. After his death, whole-exome sequencing revealed a homozygous 22,736 bp-deletion encompassing the exons 8–11 of *RIPK1* and exon 1 of the consecutive gene *BPHL* that was validated using PCR and Sanger sequencing (Fig. 3A and B). PCR and Sanger sequencing of cDNA synthesized from mRNA from a patient-derived EBV-immortalized B-cell line shows that the deletion leads to the expression of RIPK1-BPHL fusion transcripts. We could detect two differently spliced fusion transcripts, both of which are predicted to result in a frameshift and stopgain, leading to a premature stop in *RIPK1* exon 6 and *BPHL* exon 3, respectively. The fusion RIPK1-BPHL transcripts and the positions of the introduced stop codons are illustrated in the supplementary Fig. 3 C-E. This indicates that that no functional RIPK1 protein is expressed in the patient’s cells because, even if the mutant transcript were to evade nonsense-mediated decay, the predicted protein would be truncated within or shortly after the RIPK1 kinase domain.

## Discussion

Receptor-interacting serine/threonine Kinase 1 protein, encoded by *RIPK1*, is a key signalling molecule in inflammation and cell death pathways, involved in pro-inflammatory and pro-survival signalling following the activation of surface receptors, such as tumour necrosis factor (TNF) receptor 1, Toll-like receptor (TLR)-3, TLR4 and interferon receptors. TNF receptor (TNFR) stimulation recruits RIPK1 and ‘TNFR1-associated death domain protein’ (TRADD) which lead to activation of nuclear factor-κB (NF-κB) pathway through the intracelluler signalling complex. Also RIPK1 activation leads to caspase-8 activation and apoptosis [11–14].

The critical role of RIPK1 in the survival of intestinal epithelial cells has been demonstrated in RIPK1-deficient mice that had developed intestinal pathology via inhibition of caspase-8-mediated apoptosis [8, 9]. Whereas intestinal cell apoptosis seems to play a crucial role in mice, the symptoms in RIPK1 deficient humans are predominantly mediated by dysregulated immune signalling [11, 12].

Biallelic loss-of-function *RIPK1* variants, have been linked to severe immunodeficiency, early-onset inflammatory bowel disease and arthritis [7]. While impaired T- and B-cell differentiation, significant lymphopenia, and decreased production of proinflammatory cytokines such as IL-6, TNF, and IL-12 lead to immunodeficiency, active inflammasome formation and necroptosis might be related to the inflammatory component of the disease [9–12, 15]. Interestingly, recent studies show that variants which impair the caspase-8-mediated *RIPK1* cleavage, confer a gain-of-function effect, leading to the autosomal dominant cleavage-resistant *RIPK1*-induced autoinflammatory (CRIA) syndrome [15–18].

To our knowledge, 16 patients (age at onset 1 day − 4 years old age) with autosomal recessive *RIPK1* deficiency have been reported to date. They presented with colitis and recurrent infections, also some of them had polyarthritis, aphthous ulcers, and perianal disease that comparable to our patient, because of the critical role of *RIPK1* in controlling human immune and intestinal homeostasis [7–9, 15, 19].

Patients with RIPK1 deficiency were found to have increased pro-inflammatory cytokine IL-1β and decreased IL-10 secretion, a critical cytokine in regulation of the immune response in the gut [7]. Poor treatment response was reported to immunosuppressive treatments, such as azathioprine, corticosteroids, infliximab, IL-1 receptor antagonist, and also hematopoietic stem cell transplantation (HSCT). On the other hand, several patients were reported to be alive only with intravenous immunoglobulin, antifungal and antibiotic treatment. There was no relation between treatment success and age or clinical presentation of the patients [7–9, 15, 19].

The ability of HSCT to treat this disease is still unknown. Given RIPK1’s functions in regulating both immunological and epithelial responses, performing HSCT to treat these sufferers should be taken with caution since it may improve immunodeficiency [7–9, 15, 19]. Cuchet-Lourenco et al. [7] reported three patients with RIPK1 deficiency who underwent HSCT. Of them, intestinal symptoms and arthritis of the 30-month-old age patient improved but antibiotic treatment had been continued for the chronic lung disease. The older patients aged at 12-year-old and 13-year-old patient died due to multiorgan deficiency and severe disseminated infection respectively [7]. These different clinical manifestations and response to treatment might be related to genotype-phenotype correlations, variable penetrance, or secondary factors such as microbiome [8]. With the developing genetic and functional studies, various predisposing factors that may lead to enhancement of immune dysregulation can be determined in the future.

Here, we report an infant with a severe form of infantile IBD presenting with malnutrition, recurrent severe infections, polyarthritis, and perianal fistula tract. Whole-exome sequencing followed by PCR and Sanger sequencing revealed and confirmed a homozygous deletion in *RIPK1*, spanning 22.7 kb and covering the last four coding exons of *RIPK1* as well as the first exon of the adjacent *BPHL* gene (Fig. 3A**).** This deletion comprises more than half of the *RIPK1* coding sequence and leads to the expression of two differently spliced fusion transcripts between *RIPK1* and *BPHL* in a patient-derived B-cell line. Both detected fusion transcripts contain a frameshift followed by a premature stop codon in *RIPK1* exon 6 or *BPHL* exon 3, respectively. Hence, the patient is predicted to express no functional RIPK1 protein (Fig. 3C, D and E). Given that the phenotype of the patient is consistent with previous reports of RIPK1 deficiency, the deletion was considered causative. Notably, both the detection of large copy-number variants, from whole-exome data as well as their validation with PCR and Sanger is challenging. Hence, such variants might evade detection during routine diagnostic testing using exome sequencing. Our approach to design suitable primers for the validation in this patient is illustrated in Fig. 3B. To our knowledge, this is first report of a pathogenic deletion affecting these two genes. It has been suggested that bilalleic loss of *BPHL* is tolerated [20]. Therefore, an impact of the partial *BPHL* deletion on the patient’s phenotype was considered unlikely. *BHPL* encodes Biphenyl hydrolase like, a serine hydrolase that converts valacyclovir to acyclovir and valganciclovir to ganciclovir [21]. Since the patient was not given such antiviral treatment, it is unkown whether the *BPHL* exon 1 deletion might have impacted the response to treatment with these drugs. Moreover, a homozygous *MEFV* p.Glu148Gln missense variant (E148Q) was detected in our patient. *MEFV* encodes pyrin, which takes part in controlling the inflammation process, regulates IL-1b and nuclear factor kappa beta (NF-kB) activation and, inhibits Caspase-1 activation and apoptosis. Defective pyrin leads to Caspase-1 activation and excessive release of IL-1β [22, 23]. It has been reported that patients with the p.Glu148Gln substitution respond well to colchicum treatment, which reduces IL-1β production [24, 25]. Aydın F et al. [24] reported E148Q alteration leads to similar clinical findings to M694V mutations, but a milder disease and good response to colchium treatment, similar to the report of Topaloglu R et al. [26]. They concluded E148Q should be considered as a disease-causing mutation [24, 26]. On the other hand, it should be noted that a pathogenic effect of *MEFV* p.Glu148Gln is still being debated, since the variant is relatively common in the general population (with an allele frequency of approximately 0.3 in the East and South Asian population according to the Genome Aggregation Database 2.1.1) and, hence, meets the stand-alone criterion BA1 for benignity according to the ACMG variant interpretation guidelines [27]. Urgancı N et al. [28] reported heterozygous E148Q mutation was the most common MEFV gene mutation among 597 patients (2–18 years old age) diagnosed ulcerative colitis and Crohn disease, but the relation of clinical course of the diseases and mutation is still under debate. Unfortunately, we did not observe any beneficial effect of colchicum on the clinical course of the patient. Therefore, and due to the phenotype of the patient that was highly similar to previous reports, we consider the partial *RIPK1* deletion the likely cause of disease, although a modifying effect of *MEFV* p.Glu148Gln can not be ruled out.

In conclusion, despite the widespread utilization of genetic testing, it still has limitations in determining a specific etiology, which may prevent the decision on the appropriate treatment for the patients with VEO-IBD and associated immunodeficiencies. RIPK-1 protein deficiency and the possibility of larger copy-number variants evading detection during routine testing should be considered in the presence of VEO-IBD, polyarthritis, and recurrent infections.

## Data Availability

The clinical and laboratory data of the patient is available on our hospital software system.
